# Diuretic strategies in acute heart failure: a systematic review and network meta-analysis of randomized clinical trials

**DOI:** 10.1093/ehjcvp/pvaf067

**Published:** 2025-09-10

**Authors:** Antonio Cannatà, Gianluca Anastasia, Vincenzo De Marzo, Oren Caspi, Daniel Bromage, Italo Porto, Gianluigi Savarese, Theresa McDonagh, Zachary L Cox, Pietro Ameri

**Affiliations:** School of Cardiovascular Medicine & Sciences, King’s College London British Heart Foundation Centre of Excellence, London, UK; King’s College Hospital NHS Foundation Trust, London, UK; Department of Internal Medicine, University of Genova, Genova, Italy; Cardiology Department, Casa di Cura Villa Verde, Taranto, Italy; Department of Internal Medicine, University of Genova, Genova, Italy; Cardiology Department, IRCCS Casa Sollievo della Sofferenza, San Giovanni Rotondo, Italy; Heart Failure Unit, Cardiology Department, Rambam Health Care Campus and The Bruce and Ruth Rapport Faculty of Medicine, Technion—Israel Institute of Technology, Haifa, Israel; School of Cardiovascular Medicine & Sciences, King’s College London British Heart Foundation Centre of Excellence, London, UK; King’s College Hospital NHS Foundation Trust, London, UK; Department of Internal Medicine, University of Genova, Genova, Italy; Cardiac, Thoracic and Vascular Department, IRCCS Ospedale Policlinico San Martino, Genova, Italy; Department of Clinical Science and Education, Södersjukhuset; Karolinska Institutet, Stockholm, Sweden; School of Cardiovascular Medicine & Sciences, King’s College London British Heart Foundation Centre of Excellence, London, UK; King’s College Hospital NHS Foundation Trust, London, UK; Lipscomb University College of Pharmacy, Nashville, TN, USA; Department of Internal Medicine, University of Genova, Genova, Italy; Cardiac, Thoracic and Vascular Department, IRCCS Ospedale Policlinico San Martino, Genova, Italy

**Keywords:** Decongestion, Weight, Diuresis, Outcome, Kidney, Electrolyte

## Abstract

**Aims:**

Several diuretic strategies, including furosemide i.v. boluses (FB) or continuous infusion (FC), are used in acute heart failure (AHF).

**Methods and results:**

We systematically searched phase 3 randomized clinical trials (RCTs) evaluating diuretic regimens in admitted AHF patients within 48 h and irrespective of clinical stabilization. We calculated the odds ratio (OR) of FC or FB plus another diuretic (sequential nephron blockade, SNB) compared to FB alone on 24 h weight loss (WL) and worsening renal function (WRF), with a random-effects model with inverse variance weighting. Urine output, hypokalaemia, hyponatremia, and all-cause mortality/rehospitalization were secondary endpoints. In 25 selected RCTs (7149 patients, mean age 68.9 ± 8.7 years, mean left ventricular ejection fraction 38.2 ± 10.7%), FC [OR 1.55 (95% confidence interval 1.39–1.63)], FB plus tolvaptan [OR 1.57 (1.39–1.77)], FB plus SGLT2i [OR 1.23 (1.06–1.42)], and FB plus thiazide [OR 1.63 (1.37–1.94)] were associated with greater WL than FB. FB plus SGLT2i [OR 1.52 (1.19–1.94)] and FB plus acetazolamide [OR 1.81 (1.31–2.49)] were associated with WRF. FB plus thiazide was associated with both WRF [OR 1.78 (1.43–2.21)] and hypokalaemia [OR 1.69 (1.32–2.16)]. Results were consistent in sensitivity analyses considering urine output, RCTs protocol-established furosemide doses, or daily furosemide dose. Congestion/decongestion scores and clinical outcomes were reported in around 50% of RCTs. In an underpowered exploratory analysis, mortality/rehospitalization was non-significantly lower with SGLT2i [OR 0.45 (0.19–1.07)].

**Conclusion:**

FC and SNB improve surrogates of response to FB in AHF. SNB is also connoted by WRF and may induce hypokalaemia. The endpoints of diuretic RCTs should be revised and harmonized.

## Graphical Abstract

**Graphical Abstract:**
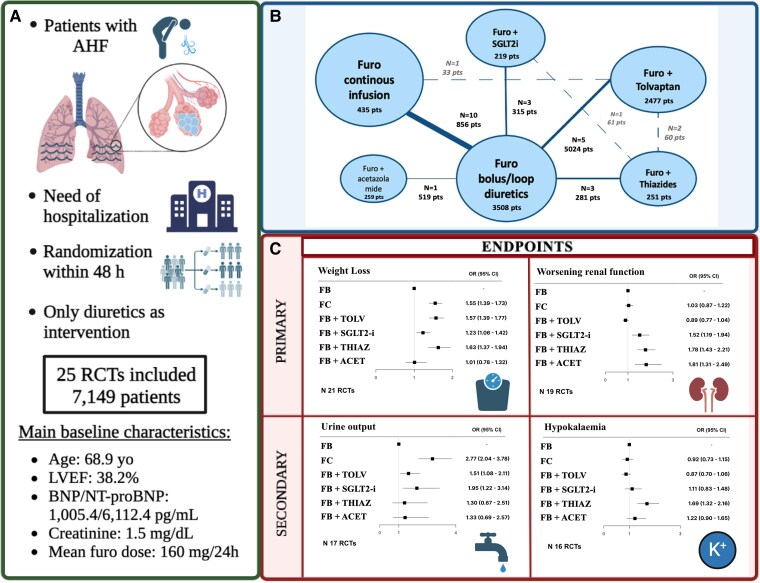

## Introduction

Intravenous (i.v.) loop diuretics (LD), administered either as bolus or as continuous infusion, are by far mostly used to treat congestion in patients with decompensated acute heart failure (AHF). To potentiate the response to LD, sequential nephron blockade (SNB) can be pursued, combining a LD with another diuretic targeting a different part of the nephron.^1^

Thiazides, such as hydrochlorothiazide and metolazone, prevent sodium reabsorption at the distal convoluted tubule.^2^ Tolvaptan, a vasopressin receptor 2 antagonist, selectively halts water resorption in the distal nephron without affecting sodium handling. Sodium-glucose cotransporter 2 inhibitors (SGLT2i) have a natriuretic effect via inhibition of glucose and sodium retention in the proximal tubule of the kidney. Acetazolamide is a carbonic anhydrase inhibitor also acting in proximal convoluted tubules, where it stimulates sodium excretion by interfering with sodium retention mediated by the luminal sodium-hydrogen exchanger 3 and basolateral sodium-bicarbonate cotransporter.

Since important randomized clinical trials (RCTs) of diuretic therapy for AHF have been performed in the last years, we sought to provide an updated quantitative synthesis of the effects of different diuretic regimens in patients admitted for AHF.

## Methods

The full methods for this systematic review and meta-analysis (PROSPERO ID 1002722) are described in the Supplemental material.

### Search strategy and data extraction

We searched the PubMed, EMBASE, SCOPUS, and/or Cochrane databases for phase 3 RCTs published between 1 January 1990 and 30 June 2023, meeting the following criteria: (i) enrolment of patients hospitalized for AHF; (ii) evaluation of the effects of LD or SNB, without other interventions (e.g. dopamine or hypertonic saline solution); (iii) randomization within 48 h from admission; (iv) no run-in period; and (v) no need of clinical stabilization before randomization.

### Endpoints

The primary endpoints were mean weight loss (WL) over 24 h of treatment and the rate of worsening renal function (WRF). Secondary endpoints were mean total urine and net urine output over 24 h (efficacy), rates of hypokalaemia and hyponatremia (safety), and all-cause mortality and/or rehospitalization.

### Statistical analysis

We compared the effects of i.v. boluses or mixed administration schemes of furosemide or other LD (overall defined as furosemide bolus, FB) with those of continuous i.v. infusion of furosemide (furosemide continuous, FC) or SNB (FB plus another diuretic). To this scope, the arms of different RCTs receiving the same diuretic therapy (e.g. FB + tolvaptan) were pooled together.

The Cochran’s Q test and Higgins and Thompsons’ *I*^2^ statistics were used to estimate heterogeneity among studies (*I*^2^ < 25%: low heterogeneity; *I*^2^ 25%–50%: moderate heterogeneity; *I*^2^ > 50%: high heterogeneity).

To improve clarity and homogenize the results, we calculated odds ratios (ORs) with 95% confidence intervals (95% CIs) by random-effects model with inverse variance weighting, as synthesis measures of the relative effect of an intervention vs. FB. Hence, OR = 1.50 for mean WL indicates that mean WL was 50% higher with the intervention than with FB, and OR = 1.50 for WRF indicates that WRF was 50% more likely with the intervention than with FB.

To investigate the influence of each study on the overall results, a leave-one-out analysis was performed for the WL and WRF endpoints. Furthermore, we calculated the ORs for WL and WRF only for those studies in which the way of administration of furosemide/LD was established per protocol, as well as for RCTs of FB vs. FC after adjusting for daily furosemide dose.

To account for furosemide dose, basal left ventricular ejection fraction (LVEF), and basal creatinine concentration, we performed a random effect meta-regression.

Risk of bias was assessed with the Cochrane Collaboration’s tool (RoB2)^3^ and publication bias was evaluated by funnel plots.

The analyses were carried out with R 4.2.1 (The R Project for Statistical Computing, Vienna) and *P* values of <0.05 were considered significant.

## Results

### Study characteristics

**Figure 1:**
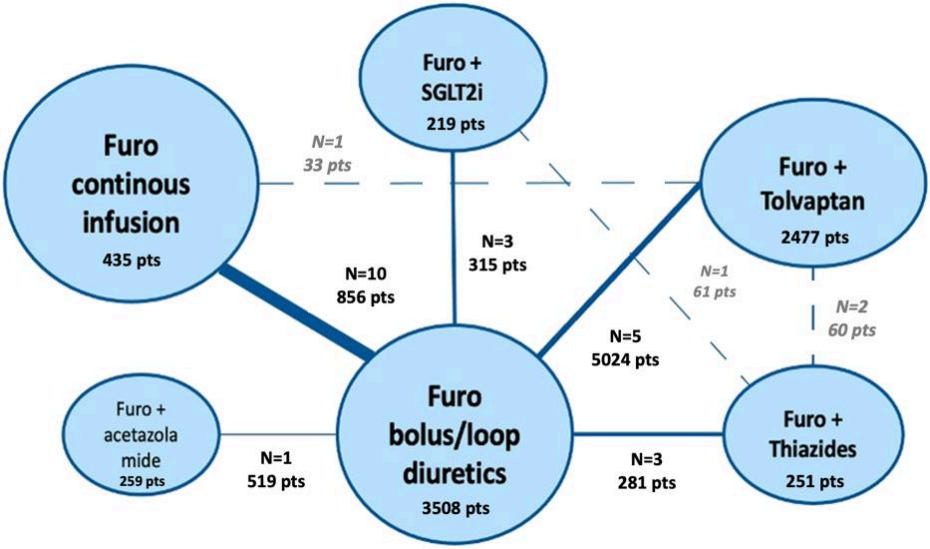
Treatment arms network plot. The solid lines indicate the diuretic regimens that were compared in the meta-analysis, with the number of included studies (*N*) and patients (pts). The number of patients in each treatment arm is also reported. SGLT2i, sodium-glucose cotransporter 2 inhibitor.

Twenty-five RCTs, accounting for 7149 patients, were included in the meta-analysis (PRISMA flow chart in Supplementary material online, *Figure S1* and PRISMA checklist in Supplementary material online, *Table S1*). Ten RCTs compared FC vs. FB,^4–13^ 12 SNB vs. furosemide/LD plus placebo,^14–25^ and 3 different strategies of SNB^25–27^ (*Figure 1*).

The main characteristics of these studies are summarized in Supplementary material online, *Table S2*. The mean age of the participants was 68.9 ± 8.7 years and the mean LVEF was 38.2 ± 10.7%. Based on four and eight studies with published information, respectively, 45% of patients had LVEF > 40% and 40% had preserved LVEF, without a specified cut-off (see Supplementary material online, *Table S3*).

Baseline concentrations of N-terminal pro-brain natriuretic peptide (NT-proBNP), BNP, creatinine, potassium, and sodium were often not reported, especially in the oldest investigations (missing: 34%, 34%, 69%, 45%, and 62%, respectively). When available, median NT-proBNP was 5287 (4690–7807) pg/mL, median BNP 1191 (790–1330) pg/mL, mean creatinine 1.5 mg/dL, mean potassium 4.2 mEq/L, and mean sodium 136 mEq/L. The median furosemide equivalent dose was 160 (95–197) mg/24 h. Doses in RCTs comparing FC vs. FB are presented in Supplementary material online, *Table S4*.

Heterogeneity among studies was moderate-to-high: 36.4% for WL, 61.9% for WRF, 44.5% for total urine output, 55.2% for net urine output, 64.7% for hypokalaemia, and 54.3% for hyponatremia. In RCTs of FC vs. FB, heterogeneity was high (52.0% for WL and 66.6% for WRF).

### Primary endpoints

**Figure 2:**
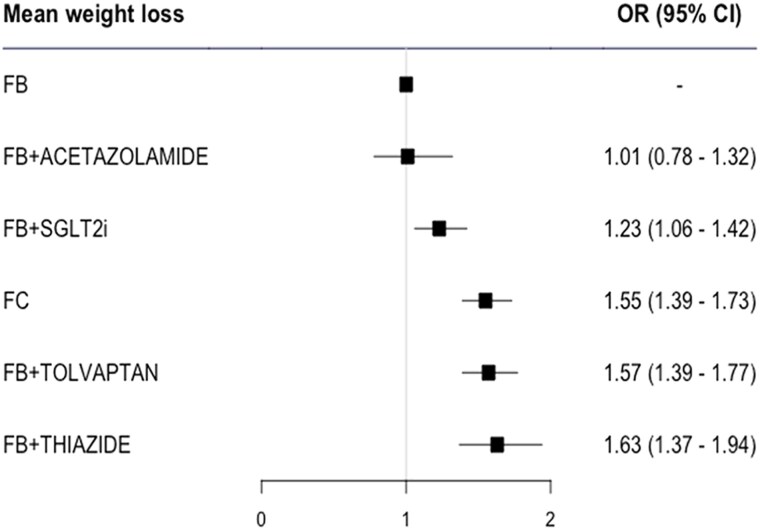
Forest plot for weight loss. FB, furosemide/loop diuretic bolus; FC, furosemide continuous; SGLT2i, sodium-glucose cotransporter 2 inhibitor. The numbers of patients in the different treatment arms were as follows: FB: 3397 patients; FB plus acetazolamide: 259 patients; FB plus SGLT2i: 219 patients; FC: 429 patients; FB plus tolvaptan: 2495 patients; FB plus thiazide: 251 patients.

A total of 6921 patients were analysed for the WL endpoint (see Supplementary material online, *Table S5*). Compared to FB, FB plus thiazide [OR 1.63 (1.37–1.94)], FB plus tolvaptan [OR 1.57 (1.39–1.77)], FC [OR 1.55 (1.39–1.63)], and FB plus SGLT2i [OR 1.23 (1.06–1.42)] were associated with greater WL (*Figure 2*).

The leave-one-out analysis confirmed the main results (see Supplementary material online, *Figure S2*).

**Figure 3:**
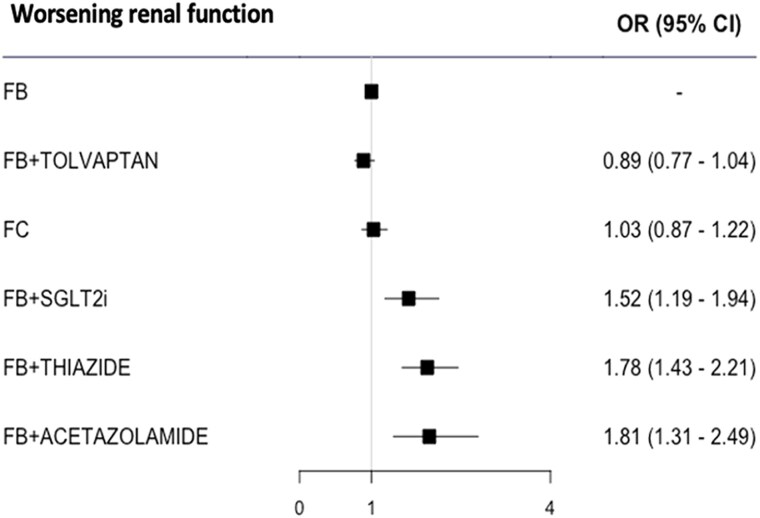
Forest plot for worsening renal function. FB, furosemide/loop diuretic bolus; FC, furosemide continuous; SGLT2i, sodium-glucose cotransporter 2 inhibitor. The numbers of patients in the different treatment arms were as follows: FB: 3415 patients; FB plus tolvaptan: 2475 patients; FC: 354 patients; FB plus SGLT2i: 219 patients; FB plus thiazide: 191 patients; FB plus acetazolamide: 259 patients.

The rate of WRF, as defined in Supplementary material online, *Table S6*, was reported in 19 (76%) RCTs and was significantly more frequent in subjects randomized to FB plus acetazolamide [OR 1.81 (1.31–2.49)], FB plus thiazide [OR 1.78 (1.43–2.21)], or FB plus SGLT2i [OR 1.52 (1.19–1.94)] than in those assigned to FB alone (*Figure 3*). The results were the same when WRF in the RCT with FB plus acetazolamide was defined as creatinine increase > 0.3 mg/dL instead of creatinine doubling, eGFR decrease > 50%, or dialysis^28^ (see Supplementary material online, *Figure S3*) and in the leave-one-out analysis (see Supplementary material online, *Figure S4*).

An additional sensitivity analysis, taking into account only 13 studies explicitly describing the furosemide/LD treatment scheme, produced similar results for WL [OR 1.59 (1.14–2.22) for FB plus thiazide, 1.63 (1.06–2.49) for FB plus tolvaptan, 1.55 (1.31–1.84) for FC, and 1.24 (0.94–1.63) for FB plus SGLT2i, as compared with FB] (see Supplementary material online, *Figure S5*). WRF could be meta-analysed for only four diuretic groups: FB, FC, FB plus tolvaptan, and FB plus thiazide. The latter two were associated with higher odds of WRF than FB (see Supplementary material online, *Figure S6*).

Given the variability in furosemide dose across RCT comparing FB and FC, we performed an additional analysis on WL, adjusting the results for the daily furosemide dose. This analysis confirmed the additional WL with FC in comparison to FB [OR 1.55 (1.31–1.85), *P* < 0.0001].

### Secondary efficacy endpoints

Total urine output was assessed in 2129 patients (29.9% of the total) and net urine output/24 h in 1293 (18.1%). Compared to FB, FC [OR 2.77 (2.04–3.78)], FB plus SGLT2i [OR 1.95 (1.22–3.14)], and FB plus tolvaptan [OR 1.51 (1.08–2.11)] were associated with greater total urine output (see Supplementary material online, *Figure S7*). Both FC [OR 1.50 (1.06–2.13)] and FB plus tolvaptan [OR 1.76 (1.06–2.92)] were also associated with greater net urine output/24 h than FB (see Supplementary material online, *Figure S8*).

### Secondary safety endpoints

**Figure 4:**
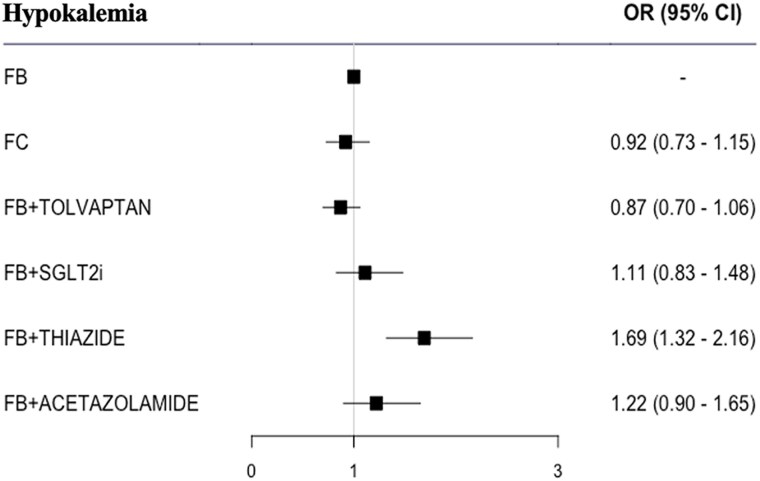
Forest plot for hypokalaemia. FB, furosemide/loop diuretic bolus; FC, furosemide continuous; SGLT2i, sodium-glucose cotransporter 2 inhibitor. The numbers of patients in the different treatment arms were as follows: FB: 2841 patients; FB plus tolvaptan: 2258 patients; FC: 305 patients; FB plus SGLT2i: 189 patients; FB plus acetazolamide: 259 patients; FB plus thiazide: 211 patients.

Hypokalaemia, based on the data from 16 (64%) RCTs, was more common in patients treated with FB plus thiazide [OR 1.69 (1.32–2.16)]; a numerically higher proportion of patients with hypokalaemia was also noted for FB plus acetazolamide (*Figure 4*).

Information about hyponatremia was retrieved for 11 (44%) RCTs, and no significant differences were revealed by the meta-analysis (see Supplementary material online, *Figure S9*).

### Mortality or rehospitalization

In-hospital or after-discharge outcomes were reported in 12 (48%) RCTs^4,6,8–11,14–17,20,23^ (*Table 1*). With the limit of an exploratory analysis, all-cause mortality or rehospitalization (11 studies, Supplementary material online, *Table S7*) was non-significantly more frequent with FB plus thiazide [OR 1.55 (0.81–2.98)] and FC [OR 1.33 (0.89–2.0)] than with FB, and non-significantly less frequent with FB plus SGLT2i [OR 0.45 (0.19–1.07)] (see Supplementary material online, *Figure S10*).

**Table 1:** Measures of decongestion and clinical outcomes in the included studies

RCT	Assessment of congestion/decongestion based on symptoms	Assessment of congestion/decongestion based on signs	Length of hospitalization	Clinical outcomes, in-hospital	Clinical outcomes, after discharge
Channer *et al*. (1994)^26^	–	–	–	–	–
Makhoul *et al*. (1997)^13^	–	CVP	–	–	–
Konstam *et al*. EVEREST (2007)^37^	Dyspnoea (dyspnoea score)	Oedema at Day 7	–	–	WHF KCCQ at Week 24 All-cause death/HFH
Allen *et al*. (2010)^6^	–	–	Yes	–	–
Thomson *et al*. (2010)^9^	–	–	Yes	–	–
Felker *et al*. DOSE (2011)^4^	Global assessment of symptoms (VAS) Dyspnoea (VAS)	Decongestion if: JVP < 8 cm H_2_O, no orthopnoea, and trace or no peripheral oedema at 72 h	–	Worsening or persistent HF	All-cause death, rehospitalization, or ER visit at 60 days Total number of days hospitalized or dead at 60 days
Lorens *et al*. (2013)^7^	Dyspnoea (Likert scale)	Orthopnoea, rales, peripheral oedema, respiratory rate, and pulse oximetry	–	–	–
Shah *et al*. (2014)^12^	–	–	Yes	–	–
Palazzuoli *et al*. (2014)^8^	–	–	–	–	All-cause death or rehospitalization at 180 days
Yayla *et al*. (2015)^10^	–	–	Yes	–	–
Matsue *et al*. AQUAMARINE (2016)^23^	Dyspnoea (Likert scale)	Orthopnoea, oedema, pulmonary congestion	Yes	–	–
Felker *et al*. TACTICS-HF (2017)^18^	Dyspnoea (Likert Scale) Dyspnoea rating from 0 to 10	Decongestion if: JVP < 8 cm H_2_O, no orthopnoea, and trace or less peripheral oedema	–	Worsening or persistent HF (need for rescue therapy)	All-cause death or rehospitalization at 30 days
Tamaki *et al*. (2017)^21^	–	–	–	–	–
Konstam *et al*. SECRET-CHF (2017)^20^	Dyspnoea (Likert scale)	–	Yes	–	All-cause death or rehospitalization at 30 days
Frea *et al*. DRAIN (2019)^5^	–	Decongestion if: JVP < 8 cm H_2_O, no orthopnoea, and trace or no peripheral oedema Wet score and need to increase diuretic therapy	–	Worsening or persistent HF	–
Damman *et al*. EMPA-RESPONSE (2020)^16^	Dyspnoea (VAS)	–	Yes	Worsening HF	All-cause death or rehospitalization at 30 and 60 days
Ng *et al*. AQUA-AHF (2020)^22^	Dyspnoea (Likert scale)	–	–	–	–
Cox *et al*. 3T Trial (2020)	–	Patient congestion score (VAS) Decongestion as assessed by the investigator	–	–	All-cause death or rehospitalization at 30 days
Zheng *et al*. (2021)^11^	Dyspnoea (Borg scale)	Decongestion if: JVP < 8 cm H_2_O, no orthopnoea, and trace or no peripheral oedema	Yes	–	–
Piardi *et al*. (2021)^17^	Dyspnoea (Likert scale) Thirst scale	Pulmonary rales, CVP, peripheral oedema, S3, and orthopnoea	Yes	–	–
Mullens *et al*. ADVOR (2022)^14^	–	Congestion score: oedema, pleural effusion, ascites	Yes	–	All-cause of death or rehospitalization at 3 months
Trullas *et al*. CLOROTIC (2022)^15^	Dyspnoea (VAS)	Rales, pleural effusion, oedema, ascites JVD, peripheral oedema	Yes	–	All-cause death or rehospitalization at 3 months
Schulze *et al*. EMPAG-HF (2022)^24^	Dyspnoea (VAS)	–	Yes	Worsening/persistent HF Survival at discharge	All-cause death at 30 days
Yeoh *et al*. (2023)^27^	–	Congestion score: oedema, pleural effusion, ascites Congestion at lung US	–	–	–
Cox *et al*. DICTATE-AHF (2024)^25^	–	Orthoedema score	Yes	Worsening HF	Rehospitalization at 30 days

CVP, central venous pressure; HF, heart failure; JVD, jugular vein distention; JVP, jugular vein pressure; VAS, visual analogic scale; WHF, worsening heart failure.

### Measures of decongestion

Reporting of measures of congestion/decongestion was inconstant and inconsistent (*Table 1*). Twelve (48%) studies had published information about symptoms of congestion, mainly dyspnoea as evaluated by means of the Likert scale, and 14 (56%) provided data about signs of congestion, with pre-specified scoring systems being adopted in 7 (28%) RCTs .^4,5,11,14,18,25,27^

### Meta-regression

Supplementary material online, *Table S8* summarizes the results of the meta-regression for furosemide dose, baseline LVEF, and baseline creatinine. A higher furosemide dose was associated with greater WL (coefficient 0.003, *P* = 0.027), but lower risk of WRF (coefficient −0.004, *P* = 0.025). LVEF was inversely associated with WL (coefficient −0.017, *P* = 0.028) and directly with WRF (coefficient 0.016, *P* = 0.008). There was also a trend for an inverse association between baseline creatinine and WRF (coefficient −1.142, *P* = 0.082).

### Risk of biases

The risk of bias for the WL endpoint was low in 14 out of 21 evaluated RCTs, while there was some concern for the remainder, mainly regarding the randomization process (see Supplementary material online, *Figure S11*). The risk of bias for the WRF endpoint was low in 14 out of 19 RCTs and intermediate in 5, again mostly because of concerns about the randomization process (see Supplementary material online, *Figure S12*). Funnel plots suggested potential publication bias (see Supplementary material online, *Figure S13*).

## Discussion

The main finding of this meta-analysis is that, in patients with AHF, FC and SNB induce more WL than FB. Although based on a smaller sample, the estimated effect on urine output was consistently greater with FC or SNB than with FB. SNB may be more associated with WRF and hypokalaemia (*Graphical abstract*).

Diuretic Optimization Strategies Evaluation (DOSE) was the largest RCT assessing FC vs. FB in AHF.^4^ However, other nine RCTs evaluated the effects of FC vs. FB.^5–13^ In agreement with prior smaller meta-analyses,^29–31^ ours shows that FC is more effective than FB, while the risk of WRF, hypokalaemia, or hyponatremia is not increased. A recent Cochrane review concluded that the evidence that FC is superior to FB is uncertain,^32^ but it focused on RCTs directly comparing FC and FB, excluding the LD arms of RCTs with SNB. Moreover, the statistical methods were partly different, in particular Rasoul *et al*. calculated the mean difference in net WL.^32^

In DOSE, high-dose furosemide led to greater WL and net fluid loss than low-dose furosemide. Consistently, we found that WL was directly proportional to furosemide amount, across the wide dose range in the examined studies. This lends support to the recommendation to properly titrate LD in AHF patients, especially if already on chronic diuretic therapy.^33^ In this regard, urinary sodium concentration is emerging as a reliable index of LD activity to guide dose escalation.^34,35^ Unfortunately, this strategy has only recently been adopted in RCTs of diuretics in AHF, thus it cannot be covered by a meta-analysis.

The reduction in body weight remained larger with FC than with FB after taking into account the daily furosemide dose. Thus, the superiority of FC cannot be merely related to administration of higher amounts of drug. Continuous infusion may allow achieving an adequate furosemide concentration more frequently and/or for longer periods.

RCT data indicate that tolvaptan is also superior to FB in promoting WL and urine output in patients with AHF, and it does not portend an increased risk of WRF or electrolyte alterations. However, it should also be noted that tolvaptan causes less natriuresis than other diuretic therapies, which is not reflected in WL or urine output. Moreover, in the Efficacy of Vasopressin Antagonism in Heart Failure Outcome Study With Tolvaptan (EVEREST),^36,37^ the clinical benefit of tolvaptan was overshadowed by the lack of impact on death and HF rehospitalization, which eventually prevented the implementation of this medication in the clinical arena.

SNB with other diuretics acting proximally (SGLT2i) or distally (thiazides) in the nephron also enhances WL in AHF. This is paralleled by WRF, which however must be interpreted in the context of decongestion and aetiology. It is now accepted that a rise in creatinine going alongside clinical improvement is a marker of haemodynamic effects and is not problematic.^38,39^ When defined as an increase in creatinine concentration > 0.3 mg/dL, WRF is also paradoxically more likely in subjects with better renal function at baseline, even if they require lower LD doses. This is confirmed by our meta-regression. Thus, WRF in isolation should not be interpreted to favouring one combination diuretic strategy over another, particularly in the case of SGLT2i that improve outcomes in AHF.^40^

The downside of SNB may be hypokalaemia. In our analysis, the risk of this electrolyte imbalance was highest with thiazide, especially hydrochlorothiazide that was the most represented drug within this class of diuretics.

WL and urine output are surrogate and imprecise measures of decongestion. Other parameters, such as dyspnoea scores or quantification of oedema, pleural effusion, and ascites, may be clinically more valuable. Yet, these assessments were not included in many RCTs, while WL was consistently reported.

Information on length of hospitalization and in-hospital and post-discharge outcomes was also scant in the RCTs we considered. With the caveat that the evaluated investigations were underpowered for this endpoint, we observed a trend for lower risk of mortality or rehospitalization with SGLT2i, in agreement with the established positive prognostic effects of these medications even in AHF.

### Limitations

The studies we analysed had significant heterogeneity, due to variable diuretic dosing protocols and definition of the WRF endpoint, and some also had significant risk of bias, particularly regarding selection. Several had small size, to mitigate for which we used random-effects models. Not having access to individual patient data, we could only partially account for potential confounders.

Patients with advanced renal disease represent a challenging subgroup, as they are prone to diuretic resistance, but only one RCT recruited subjects with eGFR < 30 mL/min/1.73 m².^11^

Torsemide has potential advantages over furosemide, but the RCT comparing it with furosemide (TRANSFORM-HF) did not explore decongestion.^41^ We could not include either high-dose spironolactone, because information was missing about the use of lower-dose spironolactone in the placebo arm of the specific RCT (ATHENA-HF),^42^ as well as about mineralocorticoid receptor antagonist use in the arms of most other RCTs.

## Conclusion

We conclude that FC may be preferred to FB to achieve decongestion in patients admitted for AHF. Tolvaptan would be a valid option, but AHF is out of the current clinical indication. Other SNB strategies also potentiate the effects of FB, but entail a higher risk of WRF and hypokalaemia. While the former is expected in the context of aggressive decongestion, the latter deserves attention (*Table 2*).

**Table 2:** Take-home points about diuretic treatment of AHF, based on the results of this meta-analysis

FC allows achieving greater surrogate markers of decongestion than FB
SNB (i.e. the combination of LD, irrespective of the way of administration, and other diuretics) is also more effective than FB
Consider WRF in the context of the expected renal haemodynamic effects of diuretics
Monitor electrolytes during SNB
Tolvaptan would be a valid option, but it does not have clinical indication for AHF
Beyond WL and urine output, evaluate symptoms and signs!
Do not forget the ultimate goal to prevent HF rehospitalization

AHF, acute heart failure; FB, FB, furosemide/loop diuretic bolus; FC, furosemide continuous; LD, loop diuretic; SNB, sequential nephron blockade; WRF, worsening renal function.

Information on key endpoints is often missing in RCTs of diuretics for AHF, including clinical measures of decongestion, patient-reported outcomes (dyspnoea improvement, quality of life), mortality and rehospitalization. Efforts to bridge these gaps are needed, as additional studies are performed to test novel strategies, such as natriuresis-guided therapy.

## Supplementary Material

pvaf067_Supplementary_Data

## Data Availability

The data underlying this article are available in the article and in its online supplementary material.
