# Supplementary material for: Diuretic strategies in acute heart failure: a systematic review and network meta-analysis of randomized clinical trials
Source: Eur Heart J Cardiovasc Pharmacother. 2025 Sep 10;12(1):6–14. doi: 10.1093/ehjcvp/pvaf067 (PMC12862974; doi:10.1093/ehjcvp/pvaf067)
Supplement: pvaf067_Supplementary_Data [file pvaf067_supplementary_data.docx]

**Diuretic strategies in acute heart failure: a systematic review and network meta-analysis of randomized clinical trials**

Cannatà A, Anastasia G, De Marzo V, Caspi O, Bromage D, Porto I, Savarese G, McDonagh T, Cox ZL, Ameri P.

**Supplemental material**

**Full methods**

***Search strategy and selection criteria***

We performed a systematic search of phase 3 RCTs published in the PubMed, EMBASE, SCOPUS and/or Cochrane databases between January 1^st^, 1990 and June 30^st^, 2023. The search script was “acute heart failure” OR “decompensated heart failure” OR “congestive heart failure” AND “loop diuretic” OR “diuretic therapy”.

Two investigators independently selected the eligible articles written in English, and screened the bibliographies to identify additional potentially suitable manuscripts according to the following criteria: i) enrolment of patients admitted for AHF; ii) evaluation of the effects of LD or SNB, without other interventions (e.g., dopamine or hypertonic saline solution); iii) randomization within 48 hours from admission; iv) no evaluation of treatment feasibility during a run-in period; and v) no need of clinical stabilization before randomization. Discordances were resolved by consensus with involvement of two additional authors.

***Data extraction***

The extracted data were number of patients per arm, age, left ventricular ejection fraction (LVEF), baseline levels of brain natriuretic peptide (BNP)/N-terminal pro-brain natriuretic peptide (NT-proBNP), creatinine, potassium and sodium, baseline estimated glomerular filtration rate (eGFR), typer and duration of treatment, modality of administration and dose of diuretic, weight loss (WL), total urine output, net urine output (difference between fluid intake and urine output), and rates of WRF, hypokalaemia, hyponatremia, and follow-up duration. We also systematically reviewed parameters of decongestion and clinical outcomes, such as length of hospitalization and mortality.

When reachable, the corresponding authors were asked aggregate data regarding the endpoints of interest, if they were not available in the retrieved publications.

***Endpoints***

The primary endpoints were weight loss over 24 hours of treatment (WL) and worsening renal function (WRF). Secondary endpoints were mean total urine and net urine output over 24 hours (efficacy), rates of hypokalaemia and hyponatremia (safety), and all-cause mortality and/or rehospitalization. When WL or urinary output were only reported after a period longer than 24 hours, these outcomes were calculated by converting the duration of observation from hours to days (i.e., total hours / 24) and then dividing the reported WL or urinary output by the obtained number.

***Statistical analysis***

We compared the effects of iv boluses or mixed administration schemes of furosemide or other LD (overall defined as furosemide bolus, FB) with those of continuous iv infusion of furosemide (furosemide continuous, FC) or of associations of furosemide/LD and other diuretics (FB plus another diuretic). To this scope, the arms of different RCTs receiving the same diuretic therapy (e.g., FB + tolvaptan) were pooled together.

To improve clarity and homogenize the results, we report odds ratios (OR) with 95% confidence intervals (95%CIs), as synthesis measures of the relative effect of an intervention vs FB. Hence, OR = 1.50 for mean WL indicates that mean WL was 50% higher with the intervention than with FB, and OR = 1.50 for WRF indicates that WRF was 50% more likely with the intervention than with FB.

The Cochran's Q test and Higgins and Thompsons’ I2 statistics were used to estimate heterogeneity among studies (I2 <25%: low heterogeneity; I2 25–50%: moderate heterogeneity; I2 >50%: high heterogeneity). A random-effects model with inverse variance weighting was used.

To investigate the influence of each study on the overall results, a leave-one-out analysis was performed for the WL and WRF endpoints by sequentially excluding each study. Furthermore, we omitted the RCTs with unspecified furosemide/LD treatment schemes and calculated the ORs for WL and WRF only for those studies in which the way of administration of furosemide/LD was established per protocol. We also carried out a sensitivity analysis of RCTs comparing FB vs FC, with adjustment for daily furosemide dose.

To account for furosemide dose, basal left ventricular ejection fraction (LVEF), and basal creatinine concentration, we performed a random effect meta-regression.

Risk of bias was independently assessed by two different authors with the Cochrane Collaboration’s tool (RoB2) (3), and discordances were resolved involving a third investigator. Publication bias was evaluated by means of funnel plots.

The analyses were carried out with R 4.2.1 (The R Project for Statistical Computing, Vienna) and P values less than 0.05 were considered significant.

**Table S1. PRISMA checklist.**

| **Section and Topic** | **Item #** | **Checklist item** | **Location where item is reported** |
| --- | --- | --- | --- |
| **TITLE** | | |  |
| Title | 1 | Identify the report as a systematic review. | 1 |
| **ABSTRACT** | | |  |
| Abstract | 2 | See the PRISMA 2020 for Abstracts checklist. | 3 |
| **INTRODUCTION** | | |  |
| Rationale | 3 | Describe the rationale for the review in the context of existing knowledge. | 4 |
| Objectives | 4 | Provide an explicit statement of the objective(s) or question(s) the review addresses. | 4 |
| **METHODS** | | |  |
| Eligibility criteria | 5 | Specify the inclusion and exclusion criteria for the review and how studies were grouped for the syntheses. | 4 |
| Information sources | 6 | Specify all databases, registers, websites, organisations, reference lists and other sources searched or consulted to identify studies. Specify the date when each source was last searched or consulted. | 4-5 |
| Search strategy | 7 | Present the full search strategies for all databases, registers and websites, including any filters and limits used. | 4 |
| Selection process | 8 | Specify the methods used to decide whether a study met the inclusion criteria of the review, including how many reviewers screened each record and each report retrieved, whether they worked independently, and if applicable, details of automation tools used in the process. | 4 |
| Data collection process | 9 | Specify the methods used to collect data from reports, including how many reviewers collected data from each report, whether they worked independently, any processes for obtaining or confirming data from study investigators, and if applicable, details of automation tools used in the process. | 4 |
| Data items | 10a | List and define all outcomes for which data were sought. Specify whether all results that were compatible with each outcome domain in each study were sought (e.g. for all measures, time points, analyses), and if not, the methods used to decide which results to collect. | 5 |
|  | 10b | List and define all other variables for which data were sought (e.g. participant and intervention characteristics, funding sources). Describe any assumptions made about any missing or unclear information. | 5 |
| Study risk of bias assessment | 11 | Specify the methods used to assess risk of bias in the included studies, including details of the tool(s) used, how many reviewers assessed each study and whether they worked independently, and if applicable, details of automation tools used in the process. | 5 |
| Effect measures | 12 | Specify for each outcome the effect measure(s) (e.g. risk ratio, mean difference) used in the synthesis or presentation of results. | 6 |
| Synthesis methods | 13a | Describe the processes used to decide which studies were eligible for each synthesis (e.g. tabulating the study intervention characteristics and comparing against the planned groups for each synthesis (item #5)). | 5 |
|  | 13b | Describe any methods required to prepare the data for presentation or synthesis, such as handling of missing summary statistics, or data conversions. | 5 |
|  | 13c | Describe any methods used to tabulate or visually display results of individual studies and syntheses. | 5 |
|  | 13d | Describe any methods used to synthesize results and provide a rationale for the choice(s). If meta-analysis was performed, describe the model(s), method(s) to identify the presence and extent of statistical heterogeneity, and software package(s) used. | 5 |
|  | 13e | Describe any methods used to explore possible causes of heterogeneity among study results (e.g. subgroup analysis, meta-regression). | 6 |
|  | 13f | Describe any sensitivity analyses conducted to assess robustness of the synthesized results. | 5-6 |
| Reporting bias assessment | 14 | Describe any methods used to assess risk of bias due to missing results in a synthesis (arising from reporting biases). | 6 |
| Certainty assessment | 15 | Describe any methods used to assess certainty (or confidence) in the body of evidence for an outcome. | 8 |
| **RESULTS** | | |  |
| Study selection | 16a | Describe the results of the search and selection process, from the number of records identified in the search to the number of studies included in the review, ideally using a flow diagram. | 37 |
|  | 16b | Cite studies that might appear to meet the inclusion criteria, but which were excluded, and explain why they were excluded. | 6 |
| Study characteristics | 17 | Cite each included study and present its characteristics. | 6 |
| Risk of bias in studies | 18 | Present assessments of risk of bias for each included study. | 6-7 |
| Results of individual studies | 19 | For all outcomes, present, for each study: (a) summary statistics for each group (where appropriate) and (b) an effect estimate and its precision (e.g. confidence/credible interval), ideally using structured tables or plots. | 8 |
| Results of syntheses | 20a | For each synthesis, briefly summarise the characteristics and risk of bias among contributing studies. | 6,21-24 |
|  | 20b | Present results of all statistical syntheses conducted. If meta-analysis was done, present for each the summary estimate and its precision (e.g. confidence/credible interval) and measures of statistical heterogeneity. If comparing groups, describe the direction of the effect. | 6 |
|  | 20c | Present results of all investigations of possible causes of heterogeneity among study results. | 6-7 |
|  | 20d | Present results of all sensitivity analyses conducted to assess the robustness of the synthesized results. | 6-8 |
| Reporting biases | 21 | Present assessments of risk of bias due to missing results (arising from reporting biases) for each synthesis assessed. | 7-8 |
| Certainty of evidence | 22 | Present assessments of certainty (or confidence) in the body of evidence for each outcome assessed. | 8 |
| **DISCUSSION** | | |  |
| Discussion | 23a | Provide a general interpretation of the results in the context of other evidence. | 8-10 |
|  | 23b | Discuss any limitations of the evidence included in the review. | 8-10 |
|  | 23c | Discuss any limitations of the review processes used. | 10 |
|  | 23d | Discuss implications of the results for practice, policy, and future research. | 10 |
| **OTHER INFORMATION** | | |  |
| Registration and protocol | 24a | Provide registration information for the review, including register name and registration number, or state that the review was not registered. | 6 |
|  | 24b | Indicate where the review protocol can be accessed, or state that a protocol was not prepared. | 6 |
|  | 24c | Describe and explain any amendments to information provided at registration or in the protocol. | 6 |
| Support | 25 | Describe sources of financial or non-financial support for the review, and the role of the funders or sponsors in the review. | - |
| Competing interests | 26 | Declare any competing interests of review authors. | 1 |
| Availability of data, code and other materials | 27 | Report which of the following are publicly available and where they can be found: template data collection forms; data extracted from included studies; data used for all analyses; analytic code; any other materials used in the review. | All |

**Table S2. Main characteristics of the included studies**

| **RCT** | **Trial arms** | **Furosemide equivalent dose**  **(mg/24h)** | **N. of**  **patients** | **Age (years)** | **LVEF (%)** | **Basal**  **NT-proBNP**  **/ BNP (pg/mL)** | **Basal creatinine (mg/dL)** | **Basal potassium (mEq/L)** | **Basal**  **sodium (mEq/L)** |
| --- | --- | --- | --- | --- | --- | --- | --- | --- | --- |
| **Channer et al.**  **(1994)** | FB +  bendrofluazide 10 mg | 80 | 20 | NA | NA | NA | NA | NA | NA |
|  | FB +  metolazone 10 mg | 80 | 20 | NA | NA | NA | NA | NA | NA |
| **Makhoul et al.**  **(1997)** | FC | 329 | 10 | NA | NA | NA | NA | NA | NA |
|  | FB/8h | 324 | 10 | NA | NA | NA | NA | NA | NA |
| **Konstam et al. EVEREST**  **(2007)** | LD + tolvaptan 30 mg | NA | 2072 | 65.9 | 27.5 | NA | NA | NA | NA |
|  | LD + placebo | 200 | 2061 | 65.6 | 27.5 | NA | NA | NA | NA |
| **Allen et al.**  **(2010)** | FB | 162 | 21 | 58 | 39 | NA | 2.1 | NA | 141 |
|  | FC | 162 | 26 | 61 | 31 | NA | 1.8 | NA | 139 |
| **Thomson et al.**  **(2010)** | FC | 197 | 26 | 56.4 | 29 | NA / 1191 | 1.73 | 3.9 | 138 |
|  | FB | 172 | 30 | 54.6 | 24 | NA / 1422 | 1.54 | 3.91 | 130 |
| **Felker et al.**  **DOSE**  **(2011)** | FB | 197 | 156 | 66.2 | 35 | 7308 / NA | 1.5 | NA | 134 |
|  | FC | 160 | 152 | 65.8 | 35 | 7570 / NA | 1.5 | NA | 138 |
| **Lorens et al.**  **(2013)** | FC | 280 | 36 | 82 | NA | 4440 / NA | 1.0 | 4.1 | 139 |
|  | FB/8h | 100 | 37 | 83 | NA | 3417 / NA | 1.1 | 4.4 | 138 |
|  | FB/6h | 120 | 36 | 83 | NA | 4088 / NA | 1.2 | 4.3 | 139 |
| **Shah et al.**  **(2014)** | FB | 110 | 30 | 59.3 | NA | NA | 1.35 | 4.12 | 133.3 |
|  | FC | 110 | 30 | 59.3 | NA | NA | 1.38 | 4.35 | 131.2 |
| **Palazzuoli et al.**  **(2014)** | FC | 170 | 43 | 80 | 34.3 | NA / 1204 | 1.62 | 4.19 | 137.2 |
|  | FB | 160 | 39 | 79 | 35.8 | NA / 1099 | 1.52 | 4.26 | 130.0 |
| **Yayla et al.**  **(2015)** | FB | 160 | 14 | 71.7 | 41.1 | 4765 / NA | 1.1 | NA | 136.2 |
|  | FC | 160 | 15 | 65.4 | 44.8 | 3973 / NA | 0.93 | NA | 137.8 |
| **Matsue et al. AQUAMARINE**  **(2016)** | LD alone | 120 | 109 | 73 | 46.8 | NA / 729 | 1.4 | 4.2 | 140.2 |
|  | LD + tolvaptan 15 mg | 80 | 108 | 73 | 45.4 | NA / 939.3 | 1.5 | 4.3 | 140.5 |
| **Felker et al.**  **TACTICS-HF**  **(2017)** | LD + placebo | 72 | 128 | 63 | 32 | 10756 / 1461 | 1.44 | NA | 136 |
|  | LD + tolvaptan 30 mg | 71 | 129 | 66 | 34 | 9694 / 1453 | 1.48 | NA | 136 |
| **Tamaki et al.**  **(2017)** | LD + tolvaptan 30 mg | 16.4 | 26 | 79 | 60.7 | NA / 576 | 1.34 | 4 | 139 |
|  | LD | 118.9 | 24 | 75 | 59.7 | NA / 737 | 1.49 | 3.9 | 139 |
| **Konstam et al. SECRET-CHF**  **(2017)** | LD + placebo | 186 | 128 | 67 | 33 | NA / 728 | 1.7 | NA | NA |
|  | LD + tolvaptan | 138 | 122 | 70 | 35 | NA / 577 | 1.7 | NA | NA |
| **Frea et al.**  **DRAIN**  **(2019)** | FB | 216 | 40 | 58.7 | 19.2 | 6250 / NA | 1.8 | 4.05 | 130.7 |
|  | FC | 195 | 40 | 63.0 | 19.4 | 10101 / NA | 1.7 | 4.0 | 129.3 |
| **Damman et al.**  **EMPA-RESPONSE (2020)** | LD +  empagliflozin 10 mg | 320 | 40 | 79 | 36 | 4406 / NA | 1.29 | NA | 135 |
|  | LD + placebo | 300 | 39 | 73 | 37 | 6168 / NA | 1.31 | NA | 135 |
| **Ng et al.**  **AQUA-AHF**  **(2020)** | FC +  tolvaptan 30 mg | 30 | 18 | 53 | 24 | 5190 / NA | 1.14 | 4.1 | 133 |
|  | FC | 120 | 15 | 59 | 33 | 8080 / NA | 0.87 | 4.2 | 134 |
| **Cox et al.**  **3T Trial**  **(2020)** | FC +  metolazone 10 mg | 770 | 20 | 61 | 35 | NA | 2.0 | 3,9 | 139 |
|  | FC +  chlorothiazide 100 mg | 675 | 20 | 67 | 29 | NA | 2.1 | 4.0 | 138 |
|  | FC  + tolvaptan 30 mg | 770 | 20 | 58 | 27 | NA | 1.8 | 4.0 | 137 |
| **Zheng et al.**  **(2021)** | FB | 180 | 39 | 65.5 | 58.8 | NA / 1421,9 | 2.27 | 4.76 | 132.56 |
|  | FC | 180 | 42 | 67.4 | 56.1 | NA / 1385,6 | 2.3 | 4.87 | 130.72 |
| **Piardi et al.**  **(2021)** | FB + HCT 50 mg | 86 | 26 | 64 | 30 | NA | 1.23 | 4.5 | 140 |
|  | FB + placebo | 82 | 25 | 64 | 31 | NA | 1.24 | 4.5 | 139 |
| **Mullens et al.**  **ADVOR**  **(2022)** | LD + placebo | 120.0 | 260 | 78.5 | 43 | 6483 / 951 | 1.5 | - | 140 |
|  | LD +  acetazolamide 500 mg | 106.7 | 259 | 77.9 | 43 | 5600 / 804 | 1.5 | - | 139 |
| **Trullas et al. CLOROTIC**  **(2022)** | FB | 93.75 | 116 | 82 | 57 | 4330 / 994 | 1.38 | 4.20 | 140 |
|  | FB + HCT 50 mg | 85.0 | 114 | 83 | 55 | 4720 / 1468 | 1.45 | 4.37 | 130 |
| **Schulze et al.**  **EMPAG-HF**  **(2022)** | LD +  empagliflozin 25 mg | 62.6 | 30 | 72.9 | 45 | 4726 / NA | 1.21 | 4.10 | 140 |
|  | LD + placebo | 70.2 | 29 | 76.5 | 44 | 4823 / NA | 1.11 | 3.90 | 139 |
| **Yeoh et al.**  **(2023)** | LD +  dapagliflozin 10 mg | 260 | 30 | 79 | 45 | 4855 / NA | 1.48 | 4.1 | 138 |
|  | LD +  metolazone 5-10 mg | 229 | 31 | 79 | 45 | 3806 / NA | 1.48 | 4.0 | 139 |
| **Cox et al.**  **DICTATE-AHF**  **(2024)** | LD +  dapagliflozin 10 mg | 112 | 119 | 65 | 45 | 2277 / NA | - | 3.9 | 140 |
|  | LD | 160 | 119 | 64 | 35 | 2927 / NA | - | 3.9 | 139 |

BNP, brain natriuretic peptide; FB, furosemide bolus; FC, furosemide continuous; HCT, hydrochlorothiazide; LD, loop diuretic (other than furosemide); LVEF, left ventricular ejection fraction; NA, not available; NT-proBNP, N-terminal pro-brain natriuretic peptide; RCT, randomized clinical trial.

**Table S3. Left ventricular ejection fraction groups in the included studies.**

| **RCT** | **Mean LVEF (%)** | **Patients with LVEF >40% (%)** | **Patients with HFpEF (%)** |
| --- | --- | --- | --- |
| **Channer et al.**  **(1994)** | - | - | - |
| **Makhoul et al.**  **(1997)** | - | - | - |
| **Konstam et al.**  **EVEREST**  **(2007)** | 27.5 | - | - |
| **Thomson et al.**  **(2010)** | 26.5 | - | - |
| **Allen et al.**  **(2010)** | 35.0 | - | - |
| **Felker et al.**  **DOSE**  **(2011)** | 35.0 | - | - |
| **Lorens et al.**  **(2013)** | - | - | 50 |
| **Shah et al.**  **(2014)** | - | - | - |
| **Palazzuoli et al.**  **(2014)** | 35.1 | - | - |
| **Yayla et al.**  **(2015)** | 42.5 | - | - |
| **Matsue et al.**  **AQUAMARINE**  **(2016)** | 48.4 | - | 44.8 |
| **Tamaki et al.**  **(2017)** | 60 | - | - |
| **Konstam et al.**  **SECRET-CHF**  **(2017)** | 34 | - | 32 |
| **Felker et al.**  **TACTICS-HF**  **(2017)** | 33 | - | 25 |
| **Frea et al.**  **DRAIN**  **(2019)** | 19.3 | - | - |
| **Damman et al.**  **EMPA-RESPONSE**  **(2020)** | 36.5 | - | - |
| **Cox et al.**  **3T Trial**  **(2020)** | 30 | - | 23.0 |
| **Ng et al.**  **AQUA-AHF**  **(2020)** | 28.5 | 26.1 | - |
| **Zheng et al.**  **(2021)** | 57.5 | - | - |
| **Piardi et al.**  **(2021)** | 30.5 | - | - |
| **Trullas et al.**  **CLOROTIC**  **(2022)** | 56 | - | 65.3 |
| **Mullens et al.**  **ADVOR**  **(2022)** | 43 | 56.8 | - |
| **Schulze et al.**  **EMPAG-HF**  **(2022)** | 44 | - | - |
| **Yeoh et al.**  **(2023)** | 45 | 44 | - |
| **Cox et al.**  **DICTATE-AHF**  **(2024)** | 40 | 51.7 | 42.5 |

HFpEF, heart failure with preserved ejection fraction; LVEF, left ventricular ejection fraction; RCT, randomized clinical trial.

**Table S4. Doses of furosemide in studies comparing iv infusion vs boluses of furosemide/loop diuretic.**

| **RCT** | **Intervention** | **Mean furosemide dose (mg/24h)** |
| --- | --- | --- |
| **Makhoul et al.**  **(1997)** | FC | 329 |
|  | FB/8h | 324 |
| **Allen et al.**  **(2010)** | FB | 162 ± 48 |
|  | FC | 162 ± 52 |
| **Thomson et al.**  **(2010)** | FC | 197 ± 148 |
|  | FB | 172 ± 97 |
| **Felker et al.**  **DOSE**  **(2011)** | FB | 197 |
|  | FC | 160 |
| **Lorens et al.**  **(2013)** | FC | 280 |
|  | FB/8h | 100 |
|  | FB/6h | 120 |
| **Shah et al.**  **(2014)** | FB | 110 |
|  | FC | 110 |
| **Palazzuoli et al.**  **(2014)** | FC | 170 |
|  | FB | 160 |
| **Yayla et al.**  **(2015)** | FB | 160 |
|  | FC | 160 |
| **Frea et al.**  **DRAIN**  **(2019)** | FB | 216 |
|  | FC | 195 |
| **Zheng et al.**  **(2021)** | FB | 180 |
|  | FC | 180 |

FB, furosemide bolus; FC: furosemide continuous; RCT, randomized clinical trial.

**Table S5. Endpoints of interest in the included studies.**

| **RCT** | **Trial arms** | **Primary endpoints** | | | | **Secondary endpoints (efficacy)** | | **Secondary endpoints**  **(safety)** | |
| --- | --- | --- | --- | --- | --- | --- | --- | --- | --- |
|  |  | **Timing of assessment**  **(hours)** | **WL**  **(g)** | **Timing of assessment**  **(hours)** | **WRF (%)** | **Total urine output (mL)** | **Net urine output (mL)** | **Hypokalaemia (%)** | **Hyponatremia (%)** |
| **Channer et al.**  **(1994)** | FB +  bendrofluazide 10 mg | 144 | 5050 | - | - | **-** | - | - | - |
|  | FB +  metolazone 10 mg |  | 5600 |  | - | - | - | - | - |
| **Makhoul et al.**  **(1997)** | FC | 24 | - | - | - | 2833 | **-** | **-** | **-** |
|  | FB/8h |  | - |  | - | 3672.5 | **-** | **-** | **-** |
| **Konstam et al. EVEREST**  **(2007)** | LD + tolvaptan 30 mg | 24 | 1760 | 168 | 6.4 | - | - | 8.0 | - |
|  | LD + placebo |  | 970 |  | 6.8 | - | - | 9.8 | - |
| **Allen et al.**  **(2010)** | FB | 72 | 1640 | 72 | - | 5133 | - | 24 | - |
|  | FC |  | 2660 |  | - | 4894 | - | 10 | - |
| **Thomson et al.**  **(2010)** | FC | 86.4 | 6800 | 86.4 | 19 | 13413 | 7552.8 | - | - |
|  | FB | 112.5 | 5100 | 112.5 | 17 | 13851 | 7382.8 | - | - |
| **Felker et al.**  **DOSE**  **(2011)** | FB | 72 | 3084 | 72 | 17 | - | 4237 | 1 | 1 |
|  | FC |  | 3674 |  | 19 | - | 4249 | 1 | 1 |
| **Lorens et al.**  **(2013)** | FC | 24 | **-** | 24 | 16.7 | 3705 | 2900 | 36 | 8.3 |
|  | FB/8h |  | **-** |  | 13.5 | 3093 | 2700 | 13 | 10.8 |
|  | FB/6h |  | **-** |  | 16.7 | 2670 | 2300 | 8 | 8.3 |
| **Shah et al.**  **(2014)** | FB | 96 | - | **-** | - | **-** | 3316.3 | - | **-** |
|  | FC |  | **-** |  | - | **-** | 3384.5 | - | **-** |
| **Palazzuoli et al.**  **(2014)** | FC | 120 | 4100 | 120 | 22 | **-** | 11475 | **-** | **-** |
|  | FB |  | 3500 |  | 15 | **-** | 10450 | **-** | **-** |
| **Yayla et al.**  **(2015)** | FB | 48 | 4100 | - | - | - | - | - | - |
|  | FC |  | 4600 |  | - | - | - | - | - |
| **Matsue et al. AQUAMARINE**  **(2016)** | LD alone | 48 | 1990 | 48 | 27.8 | 4997.2 | 3697.9 | - | - |
|  | LD + tolvaptan 15 mg |  | 3160 |  | 24.1 | 6464.4 | 4700.1 | - | - |
| **Felker et al.**  **TACTICS-HF**  **(2017)** | LD + placebo | 48 | 1588 | 48 | 21.1 | 1419 | **-** | **-** | **-** |
|  | LD + tolvaptan 30 mg |  | 2767 |  | 30.2 | 1948 | **-** | **-** | **-** |
| **Tamaki et al.**  **(2017)** | LD + tolvaptan 30 mg | 48 | 3100 | 48 | 12 | 4002 | **-** | 0 | 0 |
|  | LD |  | 3000 |  | 42 | 3926 | **-** | 4 | 0 |
| **Konstam et al. SECRET-CHF**  **(2017)** | LD + placebo | 48 | 1890 | 72 | 31 | - | **-** | 11.2 | 3.2 |
|  | LD + tolvaptan |  | 3090 |  | 25 | - | **-** | 8.4 | 2.5 |
| **Frea et al.**  **DRAIN**  **(2019)** | FB | 72 | 10899 | 72 | 18 | 8612 | **-** | 15 | **-** |
|  | FC |  | 14895 |  | 18 | 10020 | **-** | 10 | **-** |
| **Damman et al.**  **EMPA-RESPONSE (2020)** | LD +  empagliflozin 10 mg | 96 | 2830 | **-** | 10 | 11000 | 5000 | 10 | **-** |
|  | LD + placebo |  | 2300 |  | 8 | 7500 | 2299 | 12.8 | **-** |
| **Ng et al.**  **AQUA-AHF**  **(2020)** | FC +  tolvaptan 30 mg | 24 | - | 96 | 8 | 3000 | 2050 | 0 | 46 |
|  | FC |  | - |  | 7 | 3200 | 2000 | 7 | 87 |
| **Cox et al.**  **3T Trial**  **(2020)** | FC +  metolazone 10 mg | 48 | 4600 | 48 | - | 7780 | 4600 | 15 | 10 |
|  | FC +  chlorothiazide 100 mg |  | 5800 |  | - | 8770 | 6280 | 10 | 45 |
|  | FC +  tolvaptan 30 mg |  | 4100 |  | - | 9790 | 6430 | 10 | 10 |
| **Zheng et al.**  **(2021)** | FB | 72 | 3530 | 72 | 23.0 | 3756 | - | 17.9 | 12.8 |
|  | FC |  | 4720 |  | 16.7 | 5146 | - | 23.8 | 19.1 |
| **Piardi et al.**  **(2021)** | LD + HCT | 72 | 1780 | 72 | 58 | **-** | **-** | 3.8 | 3 |
|  | LD + placebo |  | 1050 |  | 41 | **-** | **-** | 4.5 | 4 |
| **Mullens et al.**  **ADVOR**  **(2022)** | LD + placebo | 48 | 2700 | 72 | 0.8 | 4100 | - | 3.9 | 14 |
|  | LD +  acetazolamide 500 mg |  | 3300 |  | 2.7 | 4600 | - | 5.5 | 17 |
| **Trullas et al. CLOROTIC**  **(2022)** | FB | 96 | 1500 | 96 | 17.2 | 5600 | 6740.6 | 19 | 5.2 |
|  | FB + HCT 50 mg |  | 2500 |  | 46.5 | 7100 | 6689.5 | 44.7 | 8.8 |
| **Schulze et al.**  **EMPAG-HF**  **(2022)** | LD +  empagliflozin 25 mg | 120 | 4190 | 120 | 11.5 | 10775 | 3725 | - | - |
|  | LD + placebo |  | 3020 |  | 32.1 | 8650 | 1480 | - | - |
| **Yeoh et al.**  **(2023)** | LD +  dapagliflozin 10 mg | 96 | 3000 | 96 | 47 | 4002 | **-** | 50 | 17 |
|  | LD +  metolazone 5-10 mg |  | 3600 |  | 50 | 3926 | **-** | 63 | 13 |
| **Cox et al.**  **DICTATE-AHF**  **(2024)** | LD +  dapagliflozin 10 mg | 120 | 4000 | 120 | 1.7 | 8876 | **-** | 0.9 | 0.9 |
|  | LD |  | 4200 |  | 0 | 9060 | **-** | 0.9 | 0 |

FB, furosemide bolus; FC, furosemide continuous; HCT, hydrochlorothiazide; LD, loop diuretic (other than furosemide); WL, weight loss; WRF, worsening renal function; RCT, randomized clinical trial.

**Table S6. Definitions of worsening renal function, hypokalaemia and hyponatremia in the studies included in the analysis of these endpoints.**

| **RCT** | **Definition of**  **WRF** | **Definition of hypokalaemia** | **Definition of hyponatremia** |
| --- | --- | --- | --- |
| Konstam et al.  EVEREST (2007) | Creatinine increase >0.3 mg/dL | K <3.5 mEq/L | NA |
| Allen et al.  (2010) | NA | K <3.5 mEq/L | NA |
| Thomson et al.  (2010) | Creatinine increase >0.5 mg/dL | mEq of potassium needed for correction hypokalaemia | NA |
| Felker et al.  DOSE (2011) | Creatinine increase >0.3 mg/dL | NA | NA |
| Lorens et al.  (2013) | Creatinine increase >0.3 mg/dL | K <3.5 mEq/L | Na <135 mEq/L |
| Palazzuoli et al.  (2014) | Creatinine increase >0.3 mg/dL | NA | NA |
| Matsue et al.  AQUAMARINE (2016) | Creatinine increase >0.3 mg/dL | NA | NA |
| Felker et al.  TACTICS-HF (2017) | Creatinine increase >0.3 mg/dL | NA | NA |
| Tamaki et al.  (2017) | Creatinine increase >0.3 mg/dL or >50% of baseline | K <3.5 mEq/L | Na <135 mEq/L |
| Konstam et al.  SECRET-CHF (2017) | Creatinine increase >0.3 mg/dL | K <3.5 mEq/L | Na <135 mEq/L |
| Frea et al.  DRAIN (2019) | Creatinine increase >0.3 mg/dL or >50% of baseline | K <3.5 mEq/L | NA |
| Damman et al.  EMPA-RESPONSE (2020) | eGFR <20 mL/min or creatinine doubling | K <3.5 mEq/L | Na <135 mEq/L |
| Ng et al.  AQUA-AHF (2020) | Creatinine increase >0.3 mg/dL | K <3.5 mEq/L | Na <135 mEq/L |
| Cox et al.  3T Trial (2020) | NA | K <3.5 mEq/L | Na <135 mEq/L |
| Zheng et al.  (2021) | Creatinine increase >0.3 mg/dL | K <3.5 mEq/L | Na <135 mEq/L |
| Piardi et al.  (2021) | Creatinine increase >0.3 mg/dL | K <3.5 mEq/L | Na <135 mEq/L |
| Mullens et al.  ADVOR (2022) | Creatinine doubling, eGFR decrease >50%, dialysis | K <3.0 mEq/L | Na <135 mEq/L |
| Trullas et al.  CLOROTIC (2022) | Creatinine increase >0.3 mg/dL or >50% of baseline | K <3.5 mEq/L | Na <130 mEq/L |
| Schulze et al.  EMPAG-HF (2022) | Creatinine increase >0.3 mg/dL | NA | NA |
| Yeoh et al.  (2023) | Creatinine increase >0.3 mg/dL | K <3.5 mEq/L | Na <130 mEq/L |
| Cox et al.  DICTATE-AHF (2024) | Creatinine increase 200-299% of baseline | K <3.0 mEq/L | NA |

**Table S7. All-cause mortality and rehospitalization in the included studies.**

| **RCT** | **Study sample**  **(n)** | **Treatment**  **arm #1** | **Treatment arm #2** | **All-cause mortality or rehospitalization**  **in arm #1 (%)** | **All-cause mortality or rehospitalization**  **in arm #2 (%)** |
| --- | --- | --- | --- | --- | --- |
| **Konstam et al.**  **EVEREST**  **(2007)** | 2,072 | FB | FB + tolvaptan | 42 | 40.2 |
| **Felker et al.**  **DOSE**  **(2011)** | 308 | FB | FC | 44 | 44 |
| **Palazzuoli et al.**  **(2014)** | 82 | FB | FC | 23 | 58 |
| **Konstam et al.**  **SECRET-CHF**  **(2017)** | 250 | FB | FB + tolvaptan | 16 | 14 |
| **Felker et al.**  **TACTICS-HF**  **(2017)** | 257 | FB | FB + tolvaptan | 33 | 29 |
| **Damman et al.**  **EMPA-RESPONSE**  **(2020)** | 79 | FB | FB + SGLT2i | 33 | 10 |
| **Cox et al.**  **3T Trial**  **(2020)** | 60 | FB + thiazide | FB + tolvaptan | 25 | 30 |
| **Trullas et al.**  **CLOROTIC**  **(2022)** | 230 | FB | FB + thiazide | 16.4 | 23.7 |
| **Mullens et al.**  **ADVOR**  **(2022)** | 519 | FB | FB + acetazolamide | 27.8 | 29.7 |
| **Schulze et al.**  **EMPAG-HF**  **(2022)** | 59 | FB | FB + SGLT2i | 6.9 | 3.3 |
| **Cox et al.**  **DICTATE-AHF**  **(2024)** | 119 | FB | FB + SGLT2i | 7 | 6 |

FB, furosemide bolus; FC, furosemide continuous; RCT, randomized clinical trial; SGLT2i, sodium-glucose cotransporter inhibitor.

**Table S8. Meta-regression analysis.**

|  | **Estimate** | **Standard error** | **P value** | **R2** | **Adjusted R2** |
| --- | --- | --- | --- | --- | --- |
| **WL** |  |  |  |  |  |
| Furosemide dose | 0.003 | 0.001 | 0.027 | 0.322 | 0.267 |
| Baseline LVEF | -0.017 | 0.007 | 0.028 | 0.300 | 0.250 |
| Baseline creatinine | 0.081 | 0.580 | 0.891 | 0.001 | -0.076 |
| **WRF** |  |  |  |  |  |
| Furosemide dose | -0.004 | 0.002 | 0.025 | 0.277 | 0.231 |
| Baseline LVEF | 0.016 | 0.005 | 0.008 | 0.367 | 0.327 |
| Baseline creatinine | -1.142 | 0.614 | 0.082 | 0.178 | 0.126 |

LVEF, left ventricular ejection fraction; WL, weight loss; WRF, worsening renal function.

**Figure S1. PRISMA flowchart.**


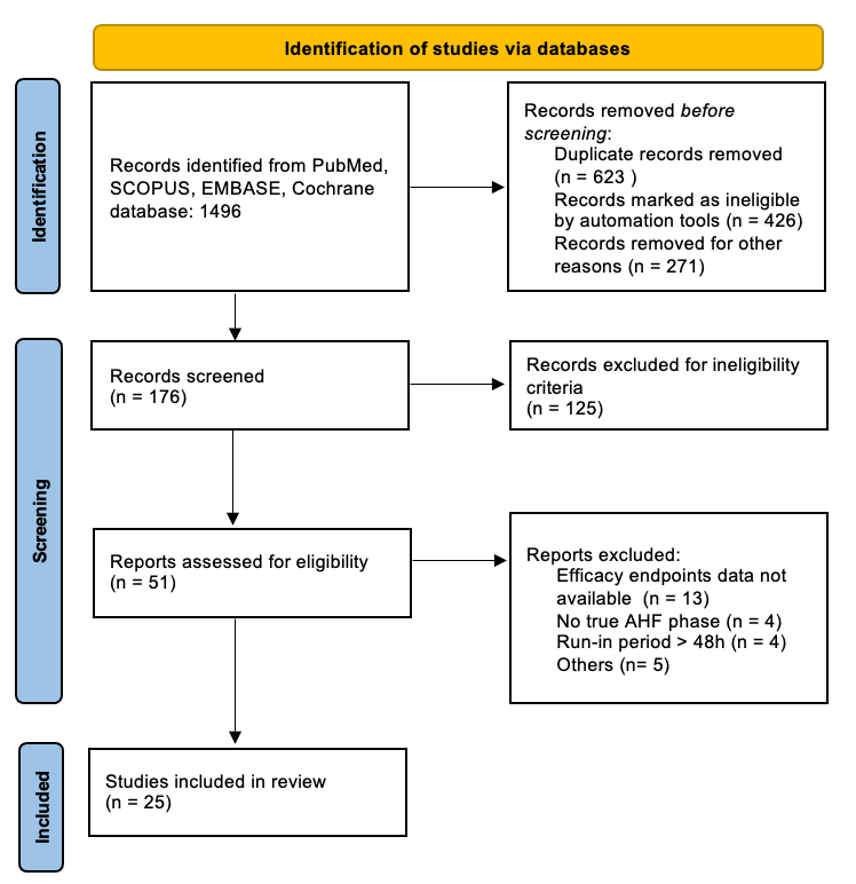


AHF, acute heart failure.

**Figure S2. Leave-one-out analysis for weight loss.**


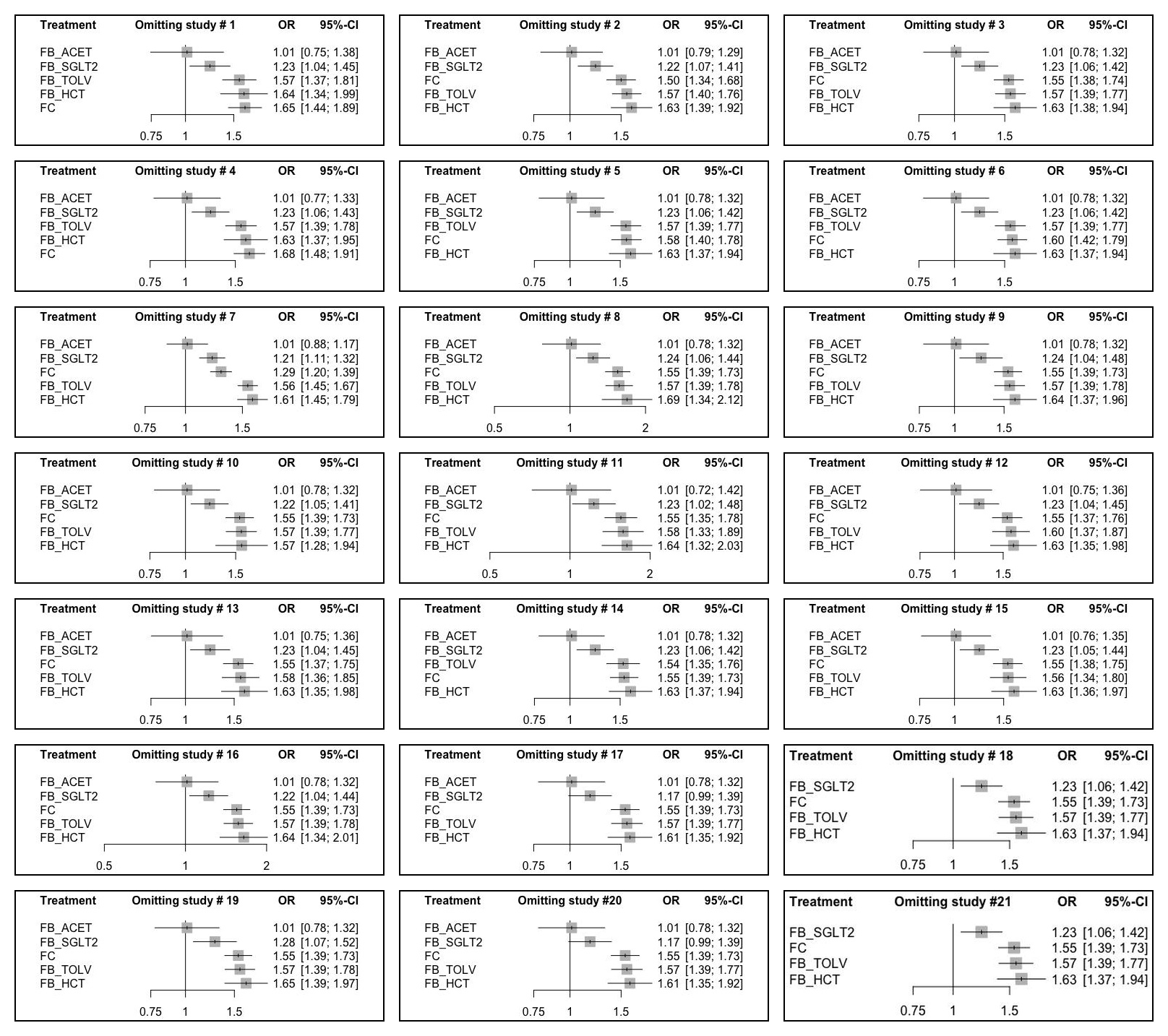


**Figure S3. Forest plot for worsening renal function, as defined by a creatinine increase >0.3 mg/dL.**


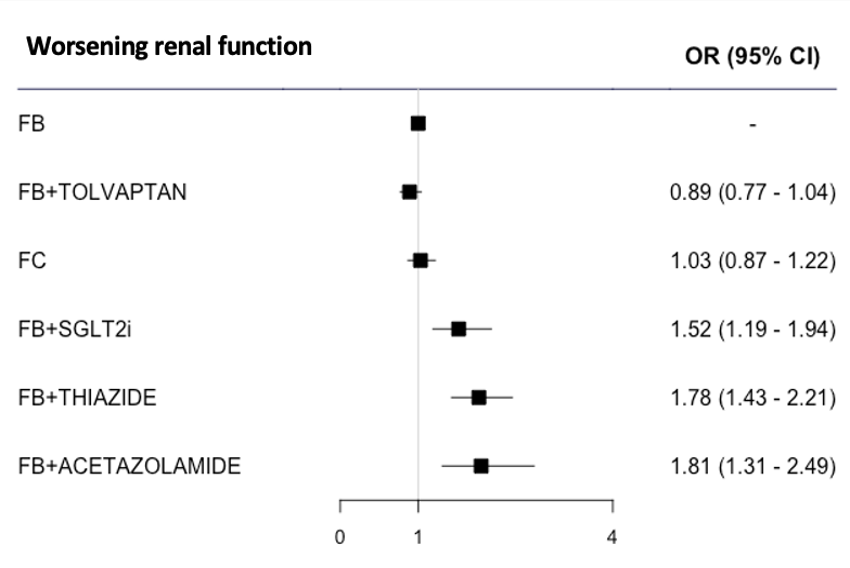


FB, furosemide/loop diuretic bolus; FC: furosemide continuous; SGLT2i, sodium-glucose cotransporter 2 inhibitor.

The numbers of patients in the different treatment arms were as follows: FB: 1,025 patients; FB plus tolvaptan: 403 patients; FC: 328 patients; FB plus SGLT2i: 149 patients; FB plus thiazide: 140 patients; FB plus acetazolamide: 259 patients.

**Figure S4. Leave-one-out analysis for worsening renal function.**


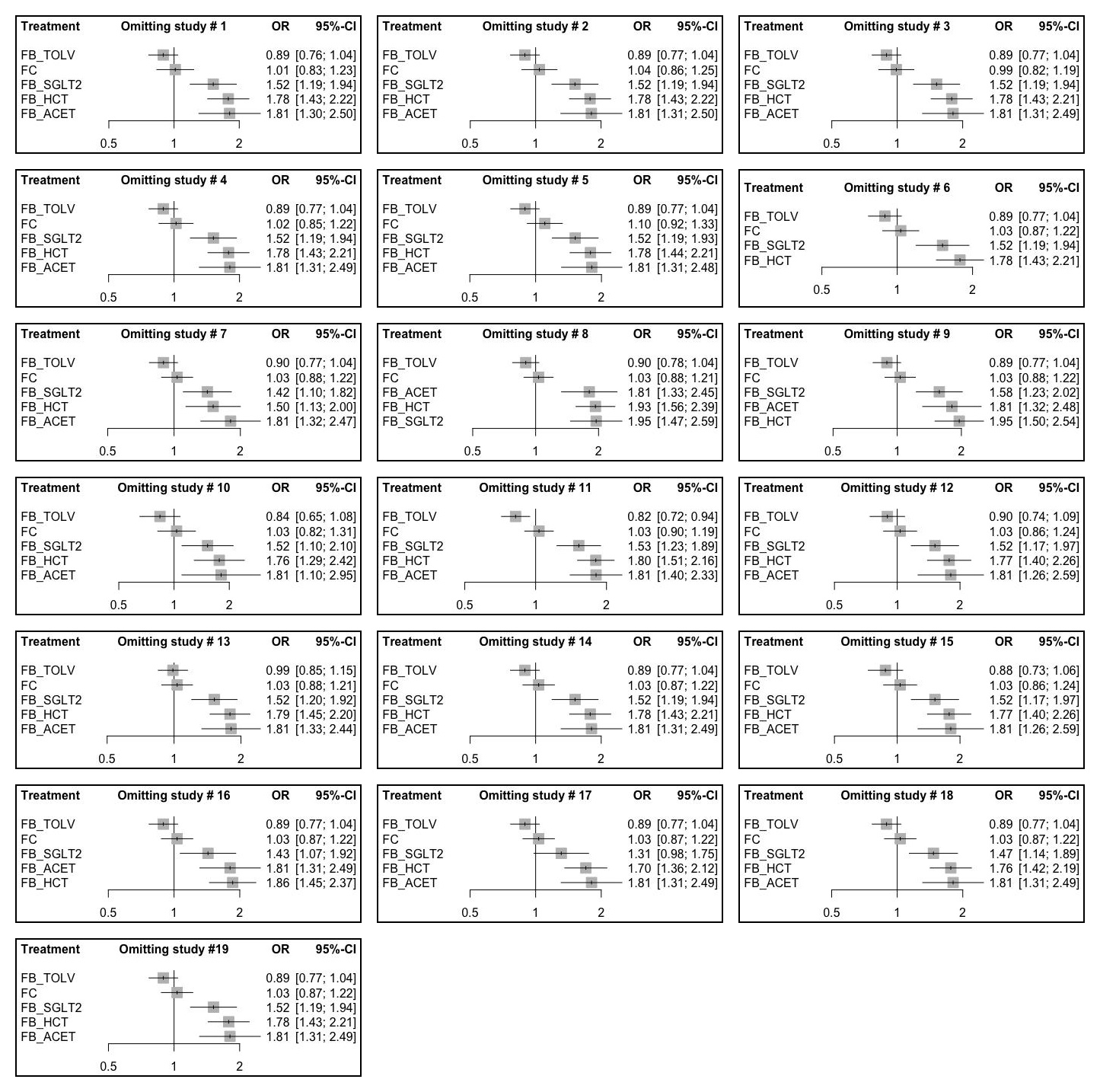


**Figure S5. Forest plot for weight loss in the studies with specified way of administration of furosemide.**

**
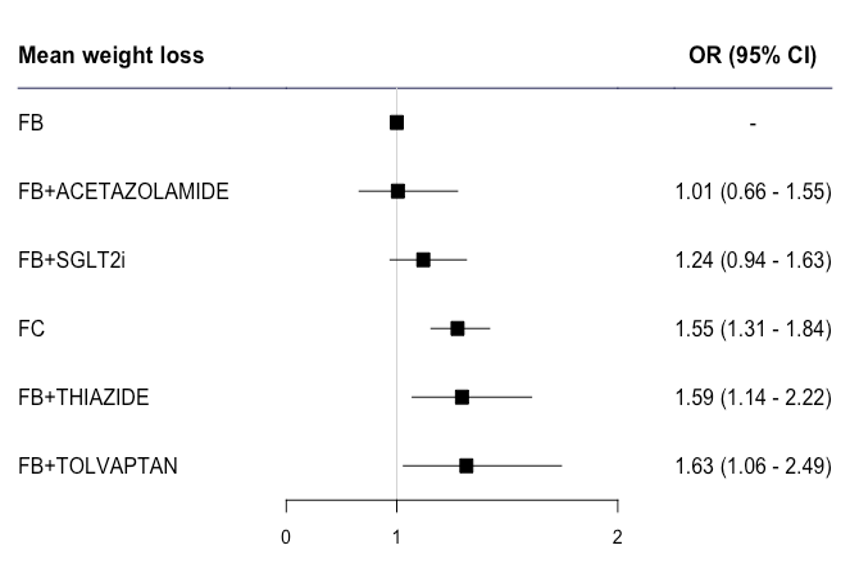
**

FB, furosemide/loop diuretic bolus; FC, furosemide continuous; SGLT2i, sodium-glucose cotransporter 2 inhibitor.

The numbers of patients in the different treatment arms were as follows: FB: 583 patients; FB plus acetazolamide: 259 patients; FB plus SGLT2i: 179 patients; FC: 338 patients; FB plus thiazide: 171 patients; FB plus tolvaptan: 405 patients.

**Figure S6. Forest plot for worsening renal function in the studies with specified way of administration of furosemide.**


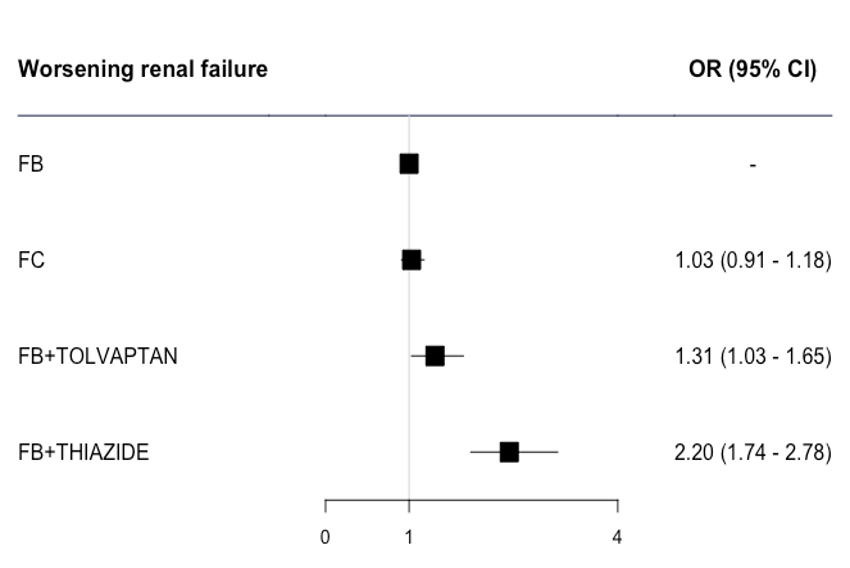


FB, furosemide/loop diuretic bolus; FC, furosemide continuous.

The numbers of patients in the different treatment arms were as follows: FB: 377 patients; FC: 354 patients; FB plus tolvaptan: 129 patients; FB plus thiazide: 114 patients.

**Figure S7. Forest plot for total urine output.**


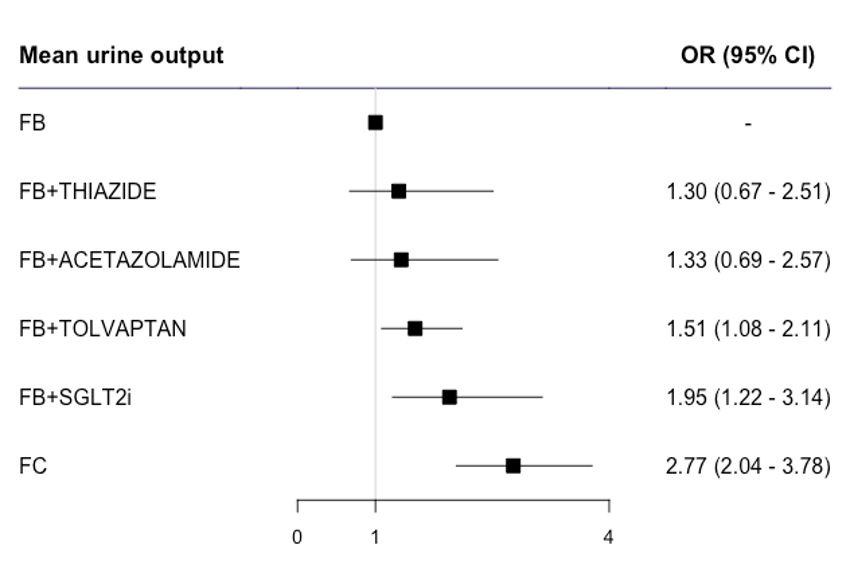


FB, furosemide/loop diuretic bolus; FC, furosemide continuous; SGLT2i, sodium-glucose cotransporter 2 inhibitor.

The numbers of patients in the different treatment arms were as follows: FB: 1,037 patients; FB plus thiazide: 154 patients; FB plus acetazolamide: 259 patients; FB plus tolvaptan: 301 patients; FB plus SGLT2i: 189 patients; FC: 189 patients.

**Figure S8. Forest plot for net urine output.**


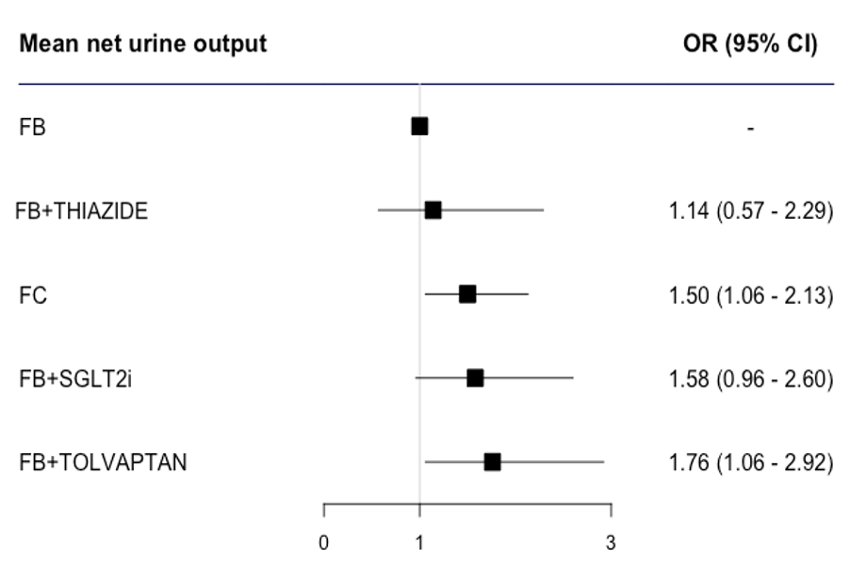


FB, furosemide/loop diuretic bolus; FC, furosemide continuous; SGLT2i, sodium-glucose cotransporter 2 inhibitor.

The numbers of patients in the different treatment arms were as follows: FB: 740 patients; FB plus thiazide: 154 patients; FC: 302 patients; FB plus SGLT2i: 169 patients; FB plus tolvaptan: 156 patients.

**Figure S9. Forest plot for hyponatremia.**


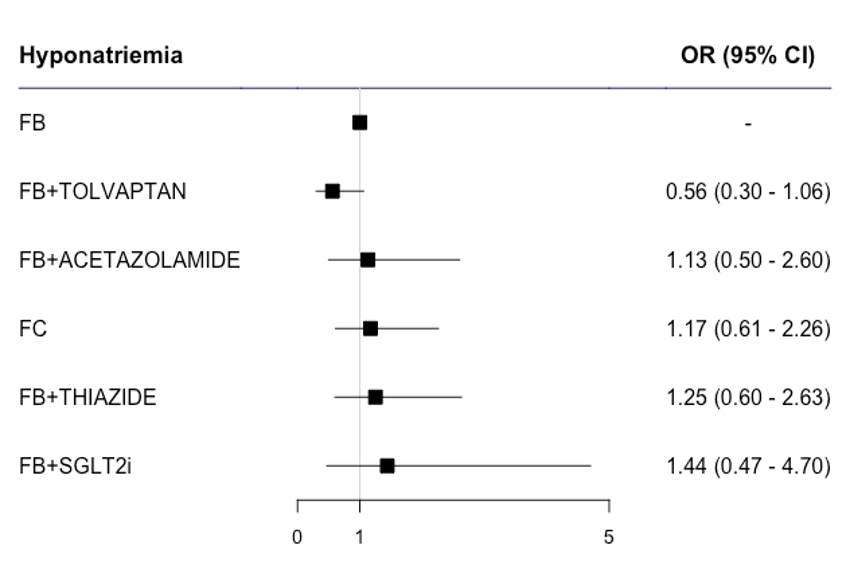


FB, furosemide/loop diuretic bolus; FC, furosemide continuous; SGLT2i, sodium-glucose cotransporter 2 inhibitor.

The numbers of patients in the different treatment arms were as follows: FB: 680 patients; FB plus tolvaptan: 186 patients; FB plus acetazolamide: 259 patients; FC: 245 patients; FB plus thiazide: 180 patients; FB plus SGLT2i: 149 patients.

**Figure S10. Forest plot for all-cause mortality or rehospitalization.**


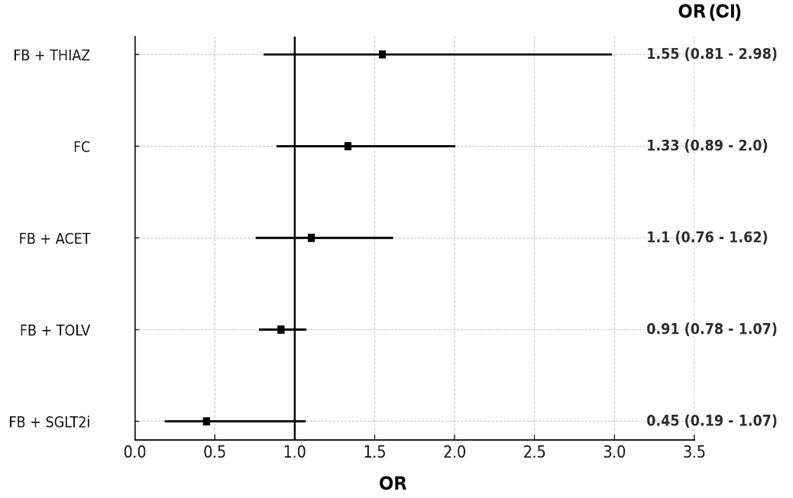


FB, furosemide/loop diuretic bolus; FC, furosemide continuous; SGLT2i, sodium-glucose cotransporter 2 inhibitor.

The numbers of patients in the different treatment arms were as follows: FB: 3073 patients; FB plus tolvaptan: 2343 patients; FB plus acetazolamide: 259 patients; FC: 195 patients; FB plus thiazide: 154 patients; FB plus SGLT2i: 189 patients.

**Figure S11. Risk of bias in the studies analysed for the weight loss endpoint.**


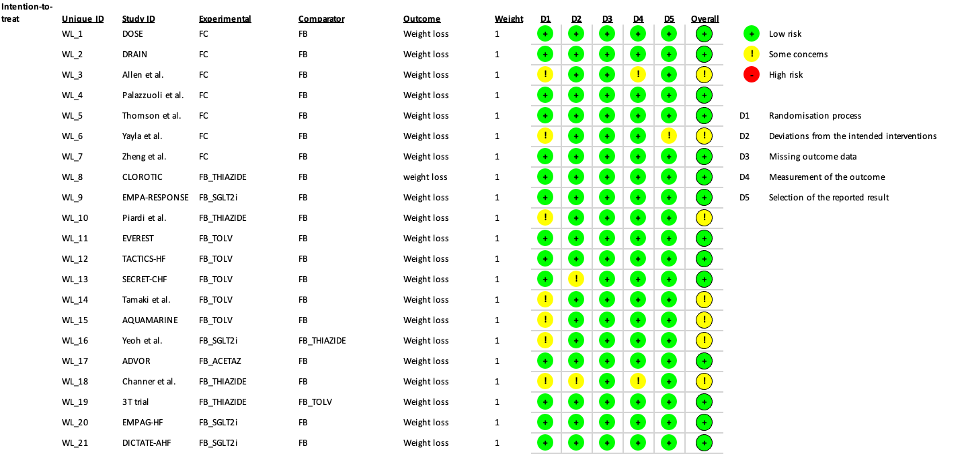


**Figure S12. Risk of bias in the studies analysed for the worsening renal function endpoint.**


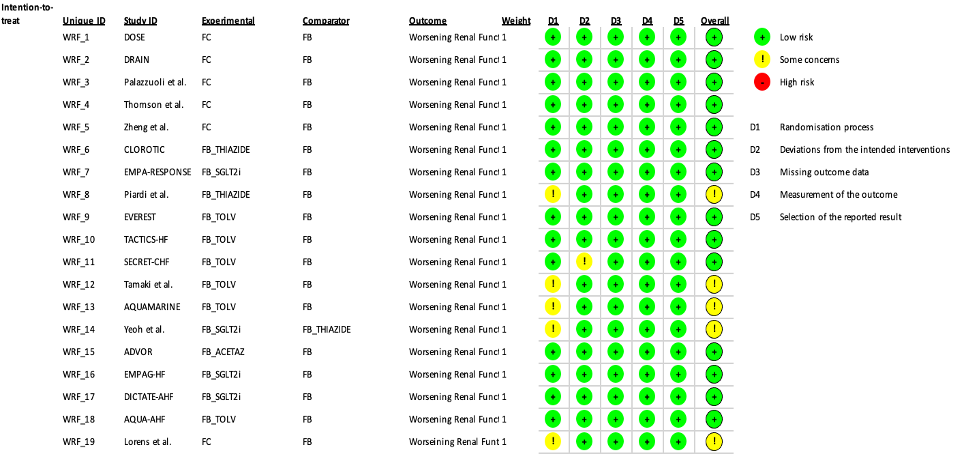


**Figure S13. Funnel plot for detection of publication bias.**

**
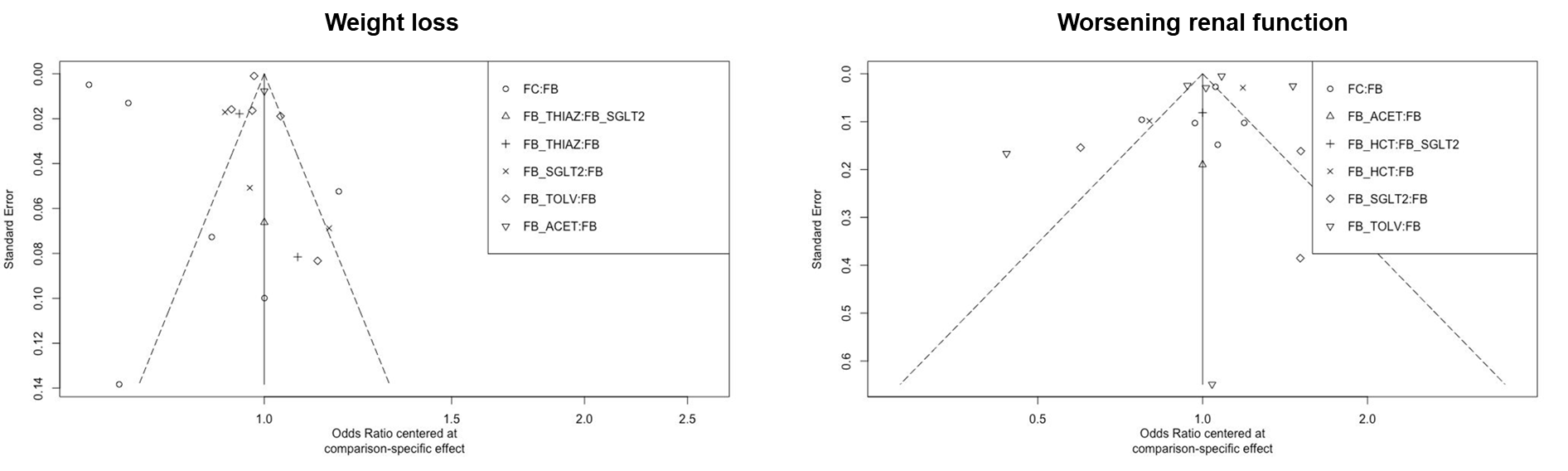
**
